# Europium (II)-Doped CaF_2_ Nanocrystals in Sol-Gel Derived Glass-Ceramic: Luminescence and EPR Spectroscopy Investigations

**DOI:** 10.3390/nano12173016

**Published:** 2022-08-31

**Authors:** Corina Secu, Arpad-Mihai Rostas, Mihail Secu

**Affiliations:** 1 National Institute of Materials Physics, Atomistilor 405A, 077125 Magurele, Romania; 2 National Institute for Research and Development of Isotopic and Molecular Technologies, Donat 67-103, 400293 Cluj-Napoca, Romania

**Keywords:** nanocrystals, glass ceramic, CaF_2_, europium, luminescence

## Abstract

The remarkable properties of Eu^2+^-activated phosphors, related to the broad and intense luminescence of Eu^2+^ ions, showed a high potential for a wide range of optical-related applications. Oxy-fluoride glass-ceramic containing Europium (II)-doped CaF_2_ nanocrystals embedded in silica matrix were produced in two steps: glass-ceramization in air at 800° with Eu^3+^-doped CaF_2_ nanocrystals embedded followed by Eu^3+^ to Eu^2+^ reduction during annealing in reducing atmosphere. The broad, blue luminescence band at 425 nm and with the long, weak tail in the visible range is assigned to the d → f type transition of the Eu^2+^ located inside the CaF_2_ nanocrystals in substitutional and perturbed sites, respectively; the photoluminescence quantum yield was about 0.76. The X-ray photoelectron spectroscopy and Electron paramagnetic spectroscopy confirmed the presence of Eu^2+^ inside the CaF_2_ nanocrystals. Thermoluminescence curves recorded after X-ray irradiation of un-doped and Eu^2+^-doped glass-ceramics showed a single dominant glow peak at 85 °C related to the recombination between F centers and Eu^2+^ related hole within the CaF_2_ nanocrystals. The applicability of the procedure can be tested to obtain an oxy-fluoride glass-ceramic doped with other divalent ions such as Sm^2+^, Yb^2+^, as nanophosphors for radiation detector or photonics-related applications.

## 1. Introduction

Rare-earth (RE) doped oxyfluoride nano-glass ceramics where the optically active RE^3+^-ions are incorporated into the precipitated fluoride nanocrystals showed high potential for optical-related applications due to their features such as high transparency and remarkable luminescence properties ([1,2] and references therein). Through a controlled nucleation and crystallization processes of the initial glass, the partition of the optically active RE^3+^-ions into the precipitated fluoride nanocrystals is obtained. Special attention was focused on optical properties of oxyfluoride nano-glass ceramics containing CaF_2_ nanocrystals, in particular doping with Eu^3+^ as a red-light luminescent ion [3,4,5,6,7,8]. It was shown that the glass-ceramic samples obtained by a melt-quenching technique showed luminescence features of both Eu^2+^ and Eu^3+^ ion species, and the Eu^3+^ ions are incorporated into the non-centrosymmetric sites of CaF_2_ nanocrystals and shows stronger emission than in the initial glass [4,5]. 

Sol-gel chemistry (using metal alkoxides and involving trifluoroacetic acid as an in-situ fluorination reagent) offers a flexible synthesis approach for the synthesis of RE^3+^-doped glass-ceramic and a wide compositional range ([9,10] and references therein). Up to now, the research efforts of oxy-fluoride glass-ceramics were focused on optical properties related to the trivalent RE^3+^-doped luminescent nanocrystals [10]. In particular, the optical properties of Eu^3+^ sol-gel derived glass-ceramic are quite similar to those obtained by melt-quenching [3,4,5] except that only the Eu^3+^ ions luminescence is observed [11,12]. Nevertheless, despite numerous studies, the sol-gel synthesis of oxy-fluorides nano-glass ceramics doped with optically active bivalent RE^2+^ -ions (such as Eu^2+^ and Sm^2+^) has not been reported in the literature. Previous investigations [13,14] have shown the incorporation of the reduced Eu^2+^ and Sm^2+^ ions in sol-gel glasses (not ceramic ones) under moderate temperature and atmospheric conditions in two steps, glass-formation and their reduction to the bivalent state by calcination in a reducing atmosphere.

The optical performances of Eu^2+^-activated phosphors have attracted significant attention because of the remarkable properties related to the broad and intense luminescence of Eu^2+^ ions. These phosphors are widely applied in various fields: lighting and display areas, scintillator detectors, X-ray storage phosphors for digital imaging applications, and persistent phosphors [15,16,17]. The optical performances are related to the broad and intense Eu^2+^ ion fluorescence, which is due to the 5d-4f parity allowing transition and is strongly dependent on the host lattice. In particular, there is an increased interest in CaF_2_:Eu^2+^ phosphor and several studies reporting various synthesis methods [18,19,20] and optical properties: scintillation, particles detection, and dosimeter properties [21,22,23]. 

Within the present study, we investigated and demonstrated the possibility to produce Europium (II)-doped CaF_2_ nanocrystals embedded in a silica matrix by using controlled reduction of Eu(III)-doped SiO_2_-CaF_2_ glass ceramics. We investigated the Eu(II) ions species and related properties using optical and magnetic resonance techniques: photoluminescence (PL) spectroscopy, quantum efficiency, thermoluminescence (TL), and electron paramagnetic resonance (EPR) spectroscopy.

## 2. Materials and Methods

### 2.1. Samples Preparation

For the preparation of the Eu^3+^(1%)-doped (94SiO_2_–5CaF_2_) (mol%) bulk xerogels, we used the sol-gel synthesis route according to the method described in Ref. [24] with reagent grade of tetraethylorthosilicate (TEOS), trifluoroacetic acid (TFA), ethyl alcohol, acetic acid (Alpha Aesar, Massachusetts, USA), and deionized water were used as starting materials. The TEOS was diluted with an equal volume of ethyl alcohol and then hydrolyzed with water under constant stirring. Calcium acetate and Europium (III) acetate hydrate were dissolved in a TFA aqueous solution. The TFA and TEOS solutions were then mixed, and acetic acid was added as a catalyst. For the TEOS:Ca(CH_3_COO)_2_:Eu(CH_3_COO)_3_∙xH_2_O:TFA:H_2_O:CH_3_COOH molar ratio, we used 19:1:0.2:3:90:3. The as-obtained sol was stirred and aged at room temperature in a sealed container, followed by drying at up to 120 °C to form the xerogel. Glass-ceramics have been obtained after annealing the dried xerogel at 800 °C for 1 h in air and subsequently in reducing atmosphere, for another hour, in 5H_2_-95Ar gas flow. After the preparation, the xerogel was clear-transparent, but, after annealing, the glass samples became milky white due to the crystallization and crushes.

### 2.2. Samples Characterization

For the thermal analysis, we have used a SETARAM Setsys Evolution 18 Thermal Analyzer (Setaram Instrumentation, Caluire-et-Cuire, France) in the 75 to 900 °C temperature range, in synthetic air (80% N_2_/20% O_2_) at a standard heating rate of 10 °C/min. Structural characterization was performed by X-ray diffractometry (XRD) and a Bruker D8 Advance type X-ray diffractometer (Billerica, MA, USA), in focusing geometry, equipped with a copper target X-ray tube and a LynxEye one-dimensional detector. The XRD pattern was recorded in the 20 to 70° range with a 0.05° step and 2 s integration time. For the phase composition and crystallographic characteristics, the XRD patterns were analyzed using the Powercell dedicated software [25]. Energy dispersive X-ray (EDX) analysis was carried out by using a Zeiss MERLIN (Jena, Germany) Compact scanning electron microscope (SEM) with a GEMINI column equipped with an energy dispersive X-ray system analyzer. The X and Q-band Electron Paramagnetic Resonance (EPR) spectroscopy measurements were carried out with a continuous-wave Elexsys 500 EPR spectrometer (Bruker AXS GmbH, Karlsruhe, Germany) equipped with a Bruker X-SHQ 4119HS-W1 X-band resonator and an ER 5106 QT-W Q-band resonator. For the X-ray photoelectron spectroscopy (XPS) measurements, we used a multianalysis SPECS system in ‘Large Area Mode’ of the XPS analyzer with very low angular acceptance, of 5° around the normal. The non-monochromatic source used an Al anode (Ex = 1486.6 eV) with an FWHM (Full Width at Half Maximum) of 0.3 eV that provided a uniform X-Ray flux on the sample surface. The electron analyzer was a PHOIBOS150 with a 150 mm radius and nine channeltron detector. The spectra were recorded with a Pass Energy of 10 eV and the extended spectra with a Pass Energy of 50 eV. In order to minimize the additional shadowing and differential charging effects, we used a dedicated flood gun Specs FG15/40.

The photoluminescence and excitation spectra were recorded at room temperature using a FluoroMax 4P spectrophotometer (HORIBA Jobin Yvon, Kyoto, Japan). We used the Quanta-Phy accessory for the quantum yield (QY) and chromaticity analysis.

## 3. Results and Discussion

### 3.1. Thermal Analysis

**Figure 1:**
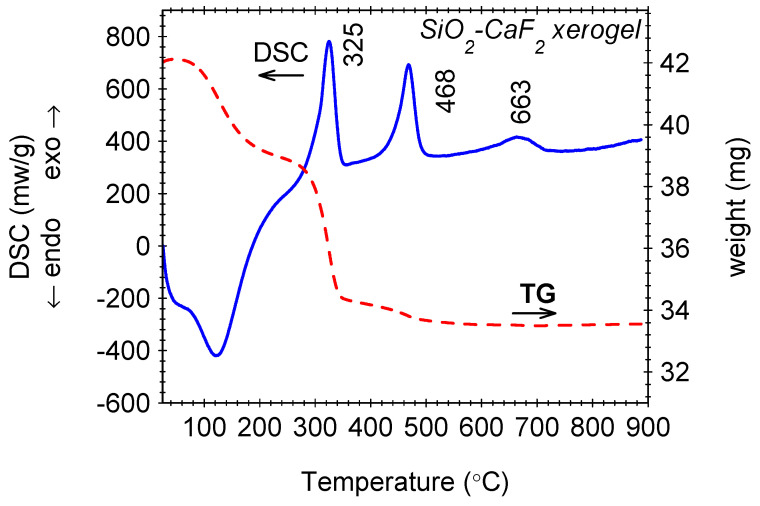
Thermal analysis results obtained on SiO_2_–CaF_2_ xerogel.

Thermogravimetry (TG) and differential scanning calorimetry (DSC) curves recorded on undoped SiO_2_-CaF_2_ xerogel (Figure 1) show a thermal degradation profile with several stages related to the glass ceramization [26]. The first one up to about 150 °C was associated with desorption of ethanol and water as well as acetic acid. A second weight loss up to about 350 °C is accompanied by a strong DSC peak at about 325 °C and is related to the Ca trifluoroacetate decomposition [24,27,28] with the formation of tiny CaF_2_ nanocrystalline seeds (a few nm size) [24,29,30]. A weaker weight loss in the 400 to 500 °C temperature range is related to the pyrolysis of organic groups. At even higher temperatures, we observed a weaker DSC peak at 663 °C, which is assigned to the initial nanocrystals’ separation, growth, and crystallinity improvement [26,28,29]. The peak assignment is consistent with its dependence on the nature of the nanocrystalline phase: at 685 °C in 95SiO_2_–5BaF_2_ [28] and 700 °C in 95SiO_2_–5SrF_2_ [30].

### 3.2. Structural Analysis

**Figure 2:**
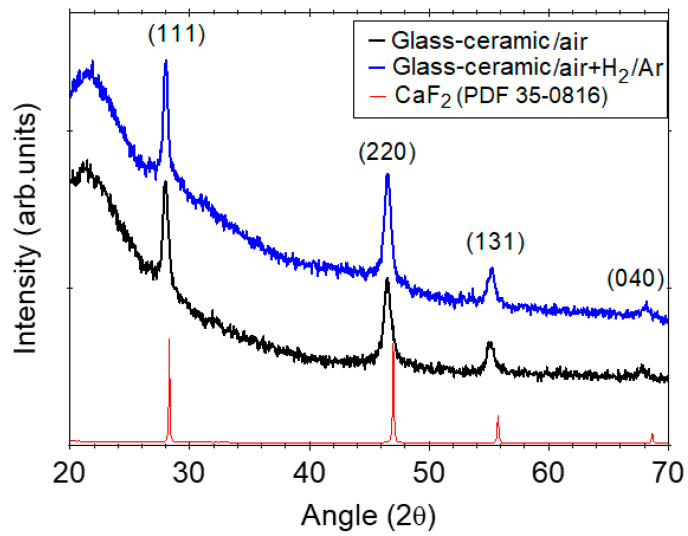
The XRD patterns of the Eu^3+^-doped SiO_2_-CaF_2_ glass-ceramics annealed in air and additionally annealed in reducing atmosphere; the XRD pattern of CaF_2_ (PDF 35–0816) is also shown.

**Figure 3:**
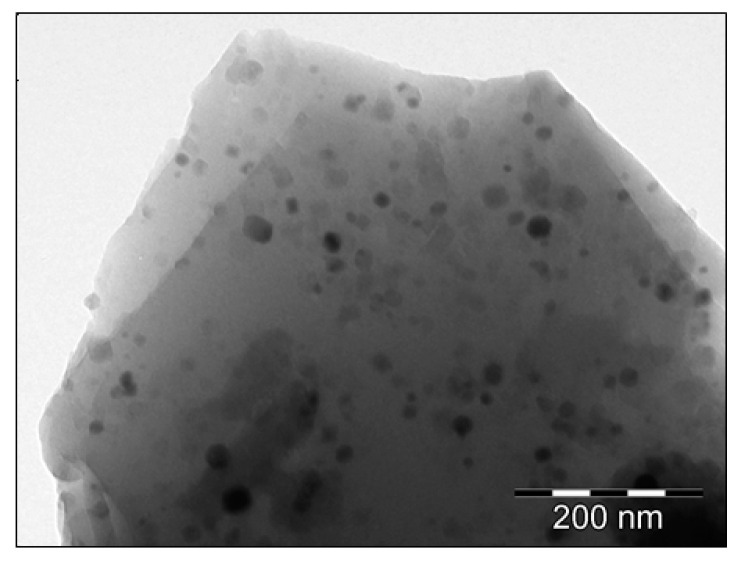
Transmission electron microscopy image of a Eu^3+^-doped SiO_2_-CaF_2_ glass-ceramic grain annealed in air (reproduced from Ref. [12]).

The XRD patterns of Eu-doped (95SiO_2_–5CaF_2_) glass-ceramics presented in Figure 2 show extra-diffraction peaks assigned to the CaF_2_ nanocrystalline cubic phase precipitation in the glass matrix superimposed on a broad background due to the amorphous silica [12]. The presence of CaF_2_ nanocrystals embedded in the glassy matrix was previously confirmed by the transmission electron microscopy (Figure 3). From the XRD pattern analysis of Eu^3+^-doped glass-ceramic (annealed in air), we extracted the lattice parameter a = 5.515 Å and the nanocrystal size of about 27 nm. The lattice parameter is different from a = 5.465 Å of the undoped glass-ceramic crystal and is consistent with the expansion of the crystalline lattice of about 1%.

The EDX spectra analysis of the glass-ceramic sample (Figure S1, supporting material) indicated the presence of elements from the precursor chemicals: 2 at%(C), 28 at%(Si), 53 at%(O), 1.5 at%(Ca), 15 at%(F), and 0.5 at%(Eu). It also indicated the presence of oxygen in the nanocrystals [31,32] that can be responsible for the lattice distortion. The incorporation of the nonbonding oxygen ions of the silica matrix [12,33] and the interstitial fluorine ions compensate for the excess positive charge caused by the Eu^3+^ doping and enlarges the crystal lattice as was observed in the nanocrystalline powders [34,35]. Hence, the ionic environment strongly influences the nanocrystal growth process in the silica matrix. Further annealing in reducing atmosphere does not change the lattice parameter a = 5.514 Å, and the nanocrystals’ mean size of about 26 nm. The nanocrystals’ size remains almost unchanged due to the interfacial interaction of SrF_2_ nano-crystals with the glass matrix, which hinders their further growth [25].

### 3.3. Optical Properties: Photoluminescence and Colorimetric Analysis

The luminescence properties of the europium ion strongly depend on the valence state (Eu^2+^ or Eu^3+^) and the matrix state, crystalline or amorphous. The Eu^3+^ luminescence is characterized by sharp peaks structured by the crystalline field (in the crystalline materials) and are assigned to the 4f → 4f transitions between various excited states and ^5^F_0_ ground state. On the other hand, Eu^2+^ luminescence has a broadband character, is strongly dependent on the host lattice, and occurs as the lowest crystal-field component of the 4f^6^5d excited configuration to the ^8^S_7/2_ ground state (parity-allowed) [36].

**Figure 4:**
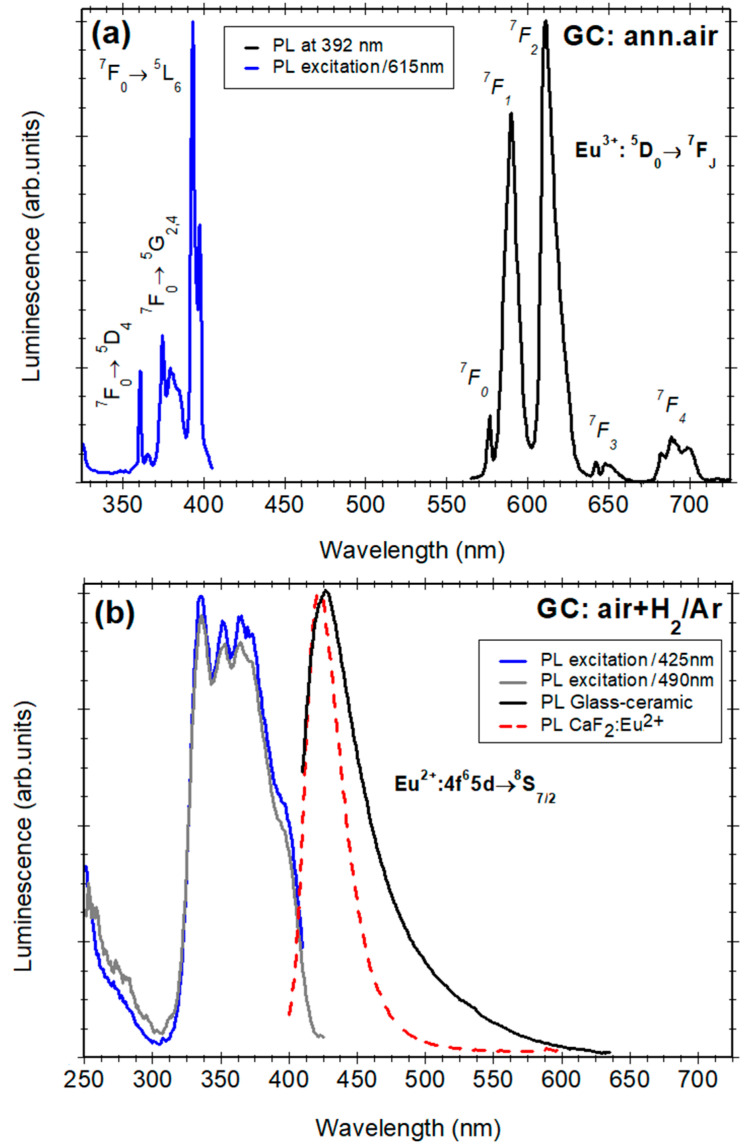
(**a**) Normalized photoluminescence spectrum recorded under 392 nm excitation and excitation spectrum of the 615 nm luminescence recorded on Eu^3+^-doped SiO_2_-CaF_2_ glass-ceramics annealed in air; (**b**) photoluminescence spectrum recorded under 392 nm (or 365 nm) excitation and the excitation spectra of 420 and 490 nm luminescence recorded on the glass-ceramics annealed in reducing atmosphere. The photoluminescence spectrum of Eu^2+^-doped CaF_2_ crystalline powder is shown for comparison (dotted curve).

The PL and PL excitation spectra of Eu^2+^/ Eu^3+^-doped SiO_2_-CaF_2_ glass-ceramic samples are presented in Figure 4. The PL spectra recorded on glass-ceramic annealed in air shows strong, sharp, and structured Eu^3+^-related luminescence peaks at 576, 590, 611, 648, and 690 nm assigned to the ^5^D_0_→^7^F_0-4_ radiative transitions accompanied by a weaker, broad, blue luminescence at about 425 nm assigned to the silica glass matrix.

The PL excitation spectrum shows several sharp peaks assigned to the intra-configurational electronic transitions of Eu^3+^ optically active ions from the ^7^F_0_ ground level to the excited states: ^5^D_4_ (363 nm), ^5^G_J_, ^5^L_7_ (372 nm–389 nm), ^5^L_6_ (392 nm). A weak shoulder at 397 nm indicated two different locations of the Eu^3+^ ions [11]. Previous investigations have shown that, in the glass-ceramic material, the Eu^3+^-ions are incorporated dominantly within the crystalline structure of the precipitated CaF_2_ nano-crystals (i.e., during the glass ceramization process); the substitution of Ca^2+^ ions by trivalent rare-earth cations leads to several different symmetries for the rare-earth sites [12,21].

The PL spectra recorded in the glass-ceramic additionally annealed in a reducing atmosphere show new features; the Eu^3+^-related luminescence peaks disappear and are replaced by a broad blue luminescence peaking at 425 nm accompanied by a weak and long tail in the visible region (Figure 4). The 425 nm luminescence band is similar to the one reported for Eu^2+^ doped CaF_2_ crystals [37]. Therefore, it was assigned to the Eu^2+^ ions that have replaced the Ca^2+^ ions in the cubic fluorite structure of the precipitated CaF_2_ nanocrystals [38]. This assumption is confirmed by the comparison between the corresponding excitation spectrum and the reported absorption spectrum [37], showing a typical "staircase" pattern between 310–425 nm, originating from transitions from the 4f^7^(^8^S_7/2_) ground state to the lowest crystal-field level of the 4f^6^5d configuration [36] from which the Eu^2+^ radiative de-excitation is observed. The excitation spectra of the 425 nm or 490 nm (luminescence on the visible tail) are quite similar, showing a broad and structured band between 310 and 410 nm due to the crystalline field splitting. The similarity indicates that the origin of the long “tail” luminescence in the visible region is related to the transitions of Eu^2+^ in a crystalline environment, i.e., the calcium fluorite structure. The visible *“tail”* luminescence indicates a second type of Eu^2+^ ions in different locations/sites inside the CaF_2_ nanoparticles with slightly perturbed coordination, supposed to be associated with some structural defects. Hence, the luminescence measurements showed that the Eu^3+^ dopant ions are reduced to their bivalent state Eu^2+^ as a consequence of the processing in the reducing atmosphere being incorporated within the CaF_2_ nanocrystaline matrix; a very small Eu^3+^ fraction might still remain in the glass matrix [12].

The nature of the perturbation affecting the Eu^2+^ luminescence is supposed to be related to the Ca^2+^ ions substitution by the trivalent rare-earth cations and the involved compensation mechanism. In the CaF_2_ crystalline structure, the excess positive charge caused by the Eu^3+^ doping is compensated by the interstitial fluorine ions or by substitutional oxygen ions in a neighboring fluorine site [38,39]. The Eu^2+^ ions species are produced due to the Eu^3+^ to Eu^2+^ reduction reaction occurring during thermal processing using the hydrogen-based reducing atmosphere: Eu^3+^ ion gains an electron from the hydrogen that loses (or “donates”) that electron and transforms to Eu^2+^. At a close look, the Eu^2+^ luminescence band is slightly broader compared to the polycrystalline powder (Figure 3) and is accompanied by the long “*tail*” in the visible region. Hence, we suppose that interstitial fluorine ions and oxygen ions (from the silica matrix [12,34]) behave as perturbation factors of the Eu^2+^ luminescence, and the broadening effect is consistent with several sites present in the nanoparticles with different site symmetries. On the other hand, the influence of the nanosize effect on the broadening of the luminescence bands cannot be neglected [12].

**Figure 5:**
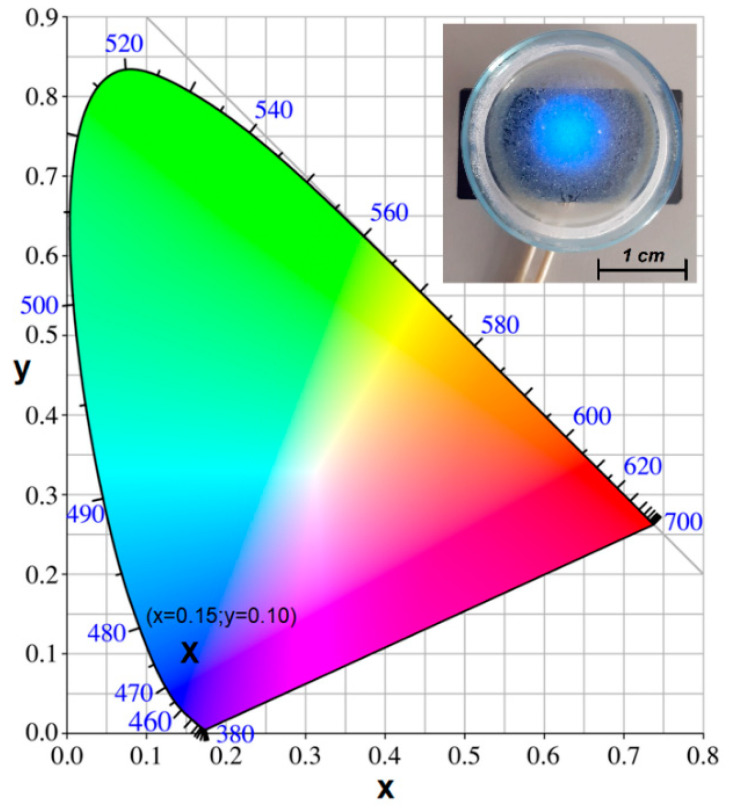
The chromaticity diagram of the Eu^2+^-doped SiO_2_-CaF_2_ glass-ceramic annealed in a reducing atmosphere showing chromaticity coordinates according to the Commission Internationale de l’Eclairage (CIE). The inset shows the image of the glass-ceramic powder annealed in a reducing atmosphere uniformly spread on the bottom of a Petri dish observed under a 345 nm UV light spot.

The additional glass-ceramic processing in a reducing atmosphere influences the color impression of the samples, and Figure 5 shows the Commission Internationale de l’Eclairage (CIE) chromaticity diagram of the Eu^2+^-doped glass-ceramic sample. Under 345 nm excitation, the glass-ceramic sample shows a strong blue color associated with the Eu^2+^ blue luminescence with the coordinates *x* = 0.15 and *y* = 0.10. The corresponding photoluminescence quantum yield was about 0.76 and is higher than for Eu^2+^-doped CaF_2_ crystal of about 0.62 at a smaller dopant concentration, below 1% mol [37].

### 3.4. Electron Paramagnetic Resonance (EPR) Analysis 

EPR spectroscopy is well-known as a powerful tool for detecting and investigating paramagnetic centers, such as Eu^2+^, as in the present material. Eu^2+^ has an S = 7/2 effective spin and two stable isotopes ^151^Eu and ^153^Eu, each with a nuclear spin number I = 5/2. In an ordered system, like a single crystal, Eu^2+^ would give rise to seven EPR lines, each split into six hyperfine lines due to the hyperfine interaction. The EPR spectra of Eu^2+^-doped crystals shows angularly dependent resonance positions, whereas, in polycrystalline media, all orientations are averaged out resulting in a complex spectrum.

**Figure 6:**
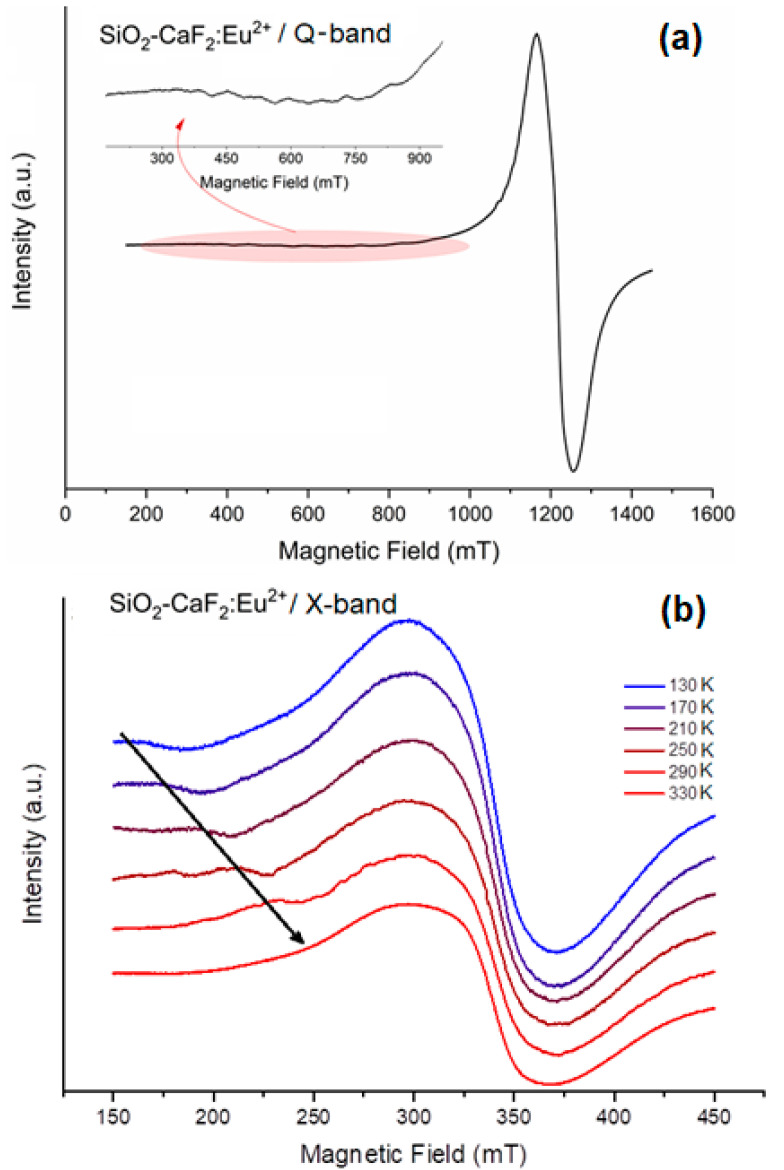
Q-band EPR spectrum of the Eu^2+^-doped SiO_2_-CaF_2_ glass-ceramic annealed in a reducing atmosphere (**a**). The inset shows a magnification of the low-field EPR resonances. X-band EPR spectra of the Eu-doped glass-ceramic annealed in a reducing atmosphere at temperatures ranging from 130 to 330 K with 40 K steps (**b**).

The Q-band EPR spectrum recorded on Eu^2+^-doped glass-ceramics annealed in a reducing atmosphere shows a comprehensive signal, with an isotropic g-value of 1.9972 and a peak-to-peak linewidth of ~91 mT (Figure 6a); this signal was not observed in the glass-ceramics annealed in air (not shown). As the spectrum is similar to that recorded on Eu^2+^ doped CaF_2_ single crystal that shows fine structure centered at g = 1.99 [40], and PL measurements showed the Eu^2+^ luminescence in doped CaF_2_ crystals, we assign the EPR signal to the Eu^2+^ ions incorporated in the precipitated CaF_2_ nano-crystals in the glassy matrix. The spectrum does not present the fine structure due to the hyperfine interaction due to the high Eu^2+^ concentration (1%), which results in a strong spin–spin exchange interaction and nanocrystals random orientation, which results in the extreme broadening effect of the EPR spectrum [41]. Nevertheless, some low-intensity resonances depicted in the inset of Figure 6a indicate the fine structure of a Eu^2+^ ion. All the observations based on EPR spectroscopy show that, after subsequent annealing of the glass-ceramic in a reducing atmosphere, the EPR *silent* Eu^3+^ ions are reduced to EPR *active* Eu^2+^.

The X-band EPR measurements were also performed on the Eu^2+^-doped glass-ceramic annealed in a reducing atmosphere at temperatures ranging from 140 to 330 K (Figure 6b), and a new EPR resonance is observed at lower temperatures in the low field region. As this resonance signal was not observed on glass-ceramics annealed in air, we supposed it to be related to the Eu^2+^ too. The signal shifts towards g~2 with increasing temperature (the arrow from the Figure 6), consistent with a strong spin–lattice and spin–spin interaction. The temperature-shifting of the EPR signal is likely caused by the changes in the T_1_ relaxation time, which is dependent on the crystalline field of the CaF_2_ host material. Therefore, we suppose that a strong effect of the CaF_2_ crystalline field is exerted on the Eu^2+^ dopant ion, and the splitting of the Eu^2+^ ground state (^8^S_7/2_) arises from higher-order perturbations involving excited states [42]. The nature of the perturbations depends on the spin–orbit and spin–spin coupling, as well as on the symmetry and magnitude of the crystalline field potential.

To summarize, the EPR signal related to the Eu^2+^ ions detected only after the glass-ceramic is annealed in a reducing atmosphere. We assign the observed broad EPR signal to the Eu^2+^ ions incorporated in the CaF_2_ nanocrystals embedded in the glass matrix. The strong spin–spin exchange interaction and nanocrystals random orientation result in the extreme broadening of the EPR spectrum.

### 3.5. X-ray Photoelectron Spectroscopy (XPS) Analysis

**Figure 7:**
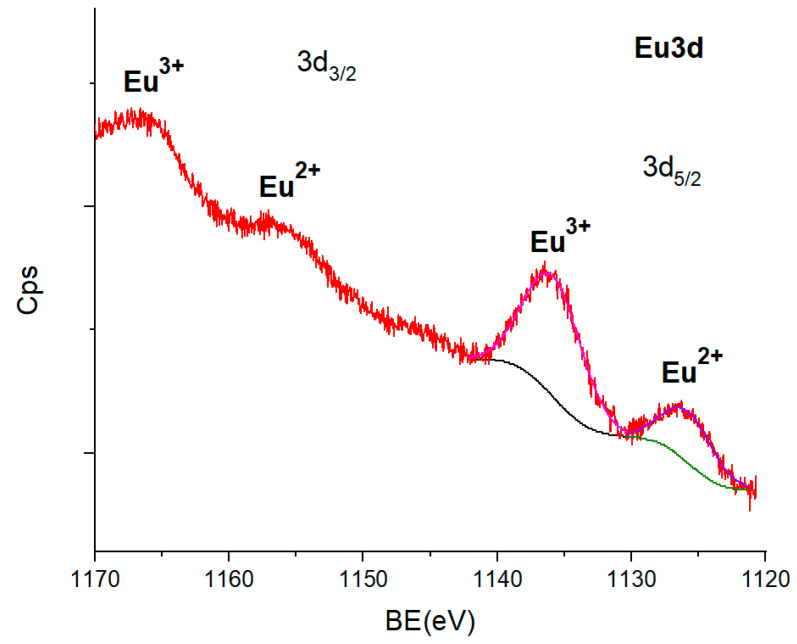
The XPS spectrum of the Eu^2+^-doped SiO_2_-CaF_2_ glass-ceramic annealed in a reducing atmosphere is shown in the Eu3d spectral line region and its deconvolution in the Eu 3d_5/2_ line region.

In order to obtain specific information about the Eu ions species and the binding energies associated with different chemical bonds, we performed an XPS analysis of the Eu^2+^-doped SiO_2_-CaF_2_ glass-ceramic annealed in a reducing atmosphere (Figure 7). 

The spectra showed the Ca-F and Si-O bonds associated with formation of CaF_2_ nanocrystals within the silica matrix, accompanied by much weaker C-C, C-O, and C-H bonds, due to the presence of calcination residue products [43] (Figure S2, supporting material). The XPS spectrum reveals interesting features in the 1120 to 1170 eV region expected for the Eu*3d* line. The spectrum is complex, composed of several convoluted peaks corresponding to the 3d_5/2_ and 3d_3/2_ Eu spin–orbit lines, separated by about 30 eV and with a peak area ratio of 3:2. For the Eu^2+^ ions, the multiplet structure is given by the two final states after photoionization, 5d^0^4f^7^ and 5d^1^4f^6^, where the satellites are relatively small, and therefore the main peaks can be revealed. As the 1125.8 eV peak energy is higher than expected for the Eu-O bond of about 1124–1125 eV, it was assigned to the Eu-F bonds [44], i.e., the Eu^2+^ incorporation within the CaF_2_ nanocrystals. The energy of the 1135.8 eV peak is slightly higher than that of the Eu_2_O_3_ oxide but smaller than for halides [45]. Therefore, it was assigned to the Eu^3+^ within an Eu-O bond, participating in a charge transfer process with surface contaminants. Hence, the Eu^3+^ ion species are present in the glass matrix, but their luminescence signal is weak, being covered by the much stronger Eu^2+^ luminescence (Figure 4). The quantification of the oxidation state of Eu is hard to do using the XPS technique (which is a thin-films investigation technique) because the Eu^2+^ ions are present in the nanocrystals, inside the volume of the glass-ceramic grain (according to the luminescence measurements), and therefore are more difficult to be observed by XPS. Hence, the ratio between Eu^3+^ and Eu^2+^ is overestimated (highly favorable to the Eu^3+^ ions), and, in this particular case, XPS provides only qualitative results.

### 3.6. Thermoluminescence (TL)

The thermoluminescence technique is a very sensitive and effective tool for investigating radiation effects in materials [46], particularly the new trapping levels induced by the RE^3+^-ions doping [47,48,49]. According to the basic model, charge carriers (electrons and holes) produced during irradiation are trapped in the band gap’s local energy levels (such as vacancies, interstitials, or impurities). During the heating, they are thermally released and recombine with carriers of the opposite sign, giving rise to TL [46]. In the present case, the effect of the reducing process of the europium ions (i.e., from Eu^3+^ to Eu^2+^) can be tracked using the thermoluminescence method too.

**Figure 8:**
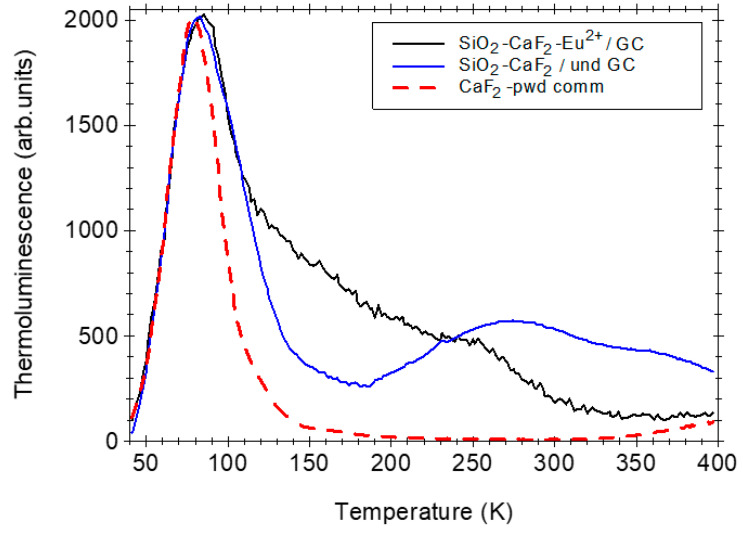
Normalized thermoluminescence curves recorded in Eu^3+^-doped SiO_2_-CaF_2_ glass-ceramics annealed in reducing atmosphere compared to those recorded in un-doped glass-ceramics annealed in air and CaF_2_ commercial crystalline powder.

In Figure 8, the TL curves recorded on Eu^2+^-doped SiO_2_-CaF_2_ glass-ceramics annealed in a reducing atmosphere are presented and compared with that recorded in undoped glass-ceramic annealed in an air atmosphere as well as with CaF_2_ commercial crystalline powder. The TL curve recorded in Eu^3+^-doped SiO_2_-CaF_2_ glass-ceramics after the calcination in air showed a dominant high-temperature peak at 370 °C (not shown) assigned to the recombination of thermally released electrons from the Eu^3+^ electron traps [12]. 

However, new features are observed after subsequent calcination in a reducing atmosphere: the glass-ceramic samples show a single dominant TL peak centered at 85 °C as in the CaF_2_ powder or at 100 °C in ceramics [21] but accompanied by broader and unresolved peaks at higher temperatures above 150 °C. Analogous with the alkali halides crystals, where the glow peaks observed above room temperature were assigned to the F-type center recombination [50], we assign the 85 °C peak to the recombination of F-centres in the CaF_2_ crystalline matrix. 

As the thermoluminescence spectra analysis of the Eu^2+^-doped CaF_2_ has shown the Eu^2+^ luminescence [21], this indicates the thermally activated recombination of F-type centers with Eu^2+^ stabilized hole centers followed by Eu^2+^ radiative emission. During the heating, the electrons are thermally released from the F-type centers and recombine with holes trapped as Eu^3+^/(Eu^2+^-hole) centers giving rise to the excited (Eu^2+^)* ions and radiative emission according to the reaction [51]:

(Eu^2+^-hole) + *é*–*k_B_T*→ (Eu^2+^)*→Eu^2+^ + *hν* (425 nm)

Hence, the effect of Eu^2+^ ions relies on stabilization of hole centers in its neighborhood by comparison with the Eu^3+^ that behaves as a deep electron trap whose recombination is observed as a TL peak at high temperatures [12,49].

At a close look, it can be seen that the 85 °C glow peak is broader than in the CaF_2_ powder ceramic [21]. The effect was assigned to the calcium fluoride lattice distortion caused by some impurities or structural defects during the nanocrystals’ growth process in the glass environment, which is strongly influenced by the ionic environment and ionic impurities [52,53]. The high-temperature TL signal above 150 °C shown by the glass-ceramic samples (but not in the crystalline powder) was observed in the quartz [54]; therefore, it was assigned to the recombination of radiation induced defects in the silica glass matrix.

A final remark about the Eu^2+^ doping effect on the TL properties can be made. Compared to the Eu^2+^-doped BaCl_2_ nanoparticles [52], where the doping has improved the TL signal by more than one order of magnitude, in the case of Eu^2+^-doped SiO_2_-CaF_2_ glass-ceramic, we observed an opposite effect, as was observed in Eu^2+^-doped CaF_2_ ceramics [21]. Both Eu^2+^-doped SiO_2_-CaF_2_ glass-ceramics and ceramics [21] showed a strong diminishing of the TL signal with more than one order of magnitude, indicating an effect related to the CaF_2_ material itself and not its morphology. Hence, through the Eu^2+^-doping, the energy of the incident X-ray radiation is converted dominantly into Eu^2+^-luminescence instead of radiation defects formation, i.e., it has improved the luminescence properties and radiation hardness by inhibiting the formation of radiation defects, in particular F-centers.

## 4. Conclusions

The sol-gel approach has been used to prepare Eu^2+^-doped CaF_2_–SiO_2_ glass-ceramics in a two-step process: the controlled crystallization at higher temperatures of the Eu^3+^-doped xerogel precursor was followed by calcination in a reducing atmosphere, under a 5H_2_-95Ar gas flow. Structural characterization using X-ray diffraction has shown CaF_2_ nanocrystals of about 27 nm in size unaffected by the subsequent calcination. The photoluminescence spectra recorded under UV-light excitation of Eu^2+^-doped glass-ceramic showed the Eu^2+^ luminescence characteristic broad bands as a consequence of the Eu^3+^ → Eu^2+^ reduction. The 425 nm luminescence and the weak, visible tail were assigned to Eu^2+^ ions inside the CaF_2_ nanocrystals in substitutional and perturbed sites, respectively. The interstitial fluorine ions and/or substitutional oxygen ions required to compensate for the Eu^3+^ ions act as perturbation factors of the Eu^2+^ luminescence and the broadening effect compared to the crystals. The X-ray photoelectron and the EPR spectra confirmed the presence of the Eu^2+^ ions inside the CaF_2_ nanocrystals and strong spin–spin exchange interaction between Eu^2+^ ions, indicating a high (>1%) doping concentration. Thermoluminescence curves of the Eu^2+^-doped glass-ceramic showed a single dominant glow peak at 85 °C due to the recombination of the F-centers and Eu^2+^ related holes within the CaF_2_ nanocrystals. The Eu^2+^-doped SiO_2_-CaF_2_ glass-ceramic obtained using the sol-gel glass technology shows high luminescence efficiency (of about 76%) and X-ray radiation hardness, which can be successfully used as novel scintillator materials for radiation detection. 

In conclusion, we demonstrated the possibility to produce Europium (II)-doped CaF_2_ nanocrystals embedded in a silica matrix by using controlled reduction of Eu(III)-doped SiO_2_-CaF_2_ glass-ceramics. The presented approach might be useful to obtain other new oxy-fluoride glass-ceramic materials doped with divalent ions such as Sm^2+^, Yb^2+^ for related applications: X-ray storage phosphor for digital imaging, persistent spectral hole burning for high-density optical memories, red broadband persistent luminescence, and white light sources.

## Data Availability

The data presented in this study are available on a reasonable request from the corresponding author.

## Supplementary Materials

The following supporting information can be downloaded at: https://www.mdpi.com/article/10.3390/nano12173016/s1, Figure S1: The EDX spectra analysis of the glass-ceramic sample; Figure S2: The XPS spectra were recorded on the glass-ceramic sample annealed in reducing atmosphere and shown in different energy regions.

## References

[1] Ming W., Jiang Z., Luo G., Xu Y., He W., Xie Z., Shen D., Li L. (2022). Transparent Nano-Glass-Ceramic for Photonic Applications. Nanomaterials.

[2] de Pablos-Martín A., Duran A., Pascual M.J. (2012). Nanocrystallisation in oxyfluoride systems: Mechanisms of crystallisation and photonic properties *Int*. Mater. Rev..

[3] Itoh M., Sakurai T., Yamakami T., Fu J. (2005). Time-resolved luminescence study of CaF_2_:Eu^2+^ nanocrystals in glass-ceramics. J. Lumin..

[4] Secu M., Secu C.E., Polosan S., Aldica G., Ghica C. (2009). Crystallization and spectroscopic properties of Eu-doped CaF_2_ nanocrystals in transparent oxyfluoride glass-ceramics. J. Non-Cryst. Solids.

[5] Jiang Y., Zhang P., Wei T., Fan J., Jiang B., Mao X., Zhang L. (2016). Europium doped transparent glass ceramics containing CaF2 micron-sized crystals: Structural and optical characterization. RSC Adv..

[6] Kemere M., Rogulis U., Sperga J. (2018). Luminescence and energy transfer in Dy^3+^/Eu^3+^ co-doped aluminosilicate oxyfluoride glasses and glass-ceramics *J*. Alloys Compd..

[7] Hu F., Zhao Z., Yin M. (2017). Structural characterization and temperature-dependent luminescence of CaF_2_:Tb^3+^/Eu^3+^ glass ceramics *J*. Rare Earths.

[8] Wang C., Chen X., Luo X., Zhao J., Qiao X., Liu Y., Fan X., Qian G., Zhang X., Han G. (2018). Stabilization of divalent Eu2+ in fluorosilicate glass ceramics via lattice site substitution. RSC Adv..

[9] Gorni G., Velázquez J.J., Mosa J., Balda R., Fernández J., Durán A., Castro Y. (2018). Transparent Glass-Ceramics Produced by Sol-Gel: A Suitable Alternative for Photonic Materials. Materials.

[10] Secu M., Secu C., Bartha C. (2021). Optical Properties of Transparent Rare-Earth Doped Sol-Gel Derived Nano-Glass Ceramics. Materials.

[11] Pawlik N., Szpikowska-Sroka B., Goryczka T., Pisarski W.A. (2019). Sol-Gel Glass-Ceramic Materials Containing CaF_2_:Eu^3+^ Fluoride Nanocrystals for Reddish-Orange Photoluminescence Applications. Appl. Sci..

[12] Secu M., Secu C.E., Ghica C. (2011). Eu^3+^-doped CaF_2_ nanocrystals in sol–gel derived glass–ceramics. Opt. Mater..

[13] Nogami M., Abe Y. (1996). Enhanced emission from Eu^2+^ ions in sol-gel derived Al_2_O_3_–SiO_2_ glasses. Appl. Phys. Lett..

[14] Nogami M., Abe Y., Hirao K., Cho D.H. (1995). Room temperature persistent spectra hole burning of Sm^2+^-doped Silicate glasses prepared by the sol-gel process. Appl. Phys. Lett..

[15] Poelman D., Smet P.F. (2011). Europium-Doped Phosphors for Lighting: The Past, the Present and the Future. International Workshop on Advanced Nanovision Science.

[16] Van den Eeckhout K., Smet P.F., Poelman D. (2010). Persistent Luminescence in Eu^2+^-Doped Compounds: A Review. Materials.

[17] Li G., Tian Y., Zhao Y., Lin J. (2015). Recent progress in luminescence tuning of Ce^3+^ and Eu^2+^-activated phosphors for pc-WLEDs. Chem. Soc. Rev..

[18] Ye W., Liu X., Huang Q., Zhou Z., Hu G. (2016). Co-precipitation synthesis and self-reduction of CaF_2_:Eu^2+^ nanoparticles using different surfactants. Mater. Res. Bull..

[19] Anghel S., Golbert S., Meijerink A., Anja–Verena M. (2017). Divalent Europium doped CaF_2_ and BaF_2_ nanocrystals from ionic liquids. J. Lumin..

[20] Ye W., Huang Q., Jiao X., Liu X., Hu G. (2017). Plasmon-enhanced fluorescence of CaF_2_:Eu^2+^ nanocrystals by Ag nanoparticles. J. Alloys Compd..

[21] Nakamura F., Kato T., Okada G., Kawaguchi N., Fukuda K., Yanagida T. (2017). Scintillation and dosimeter properties of CaF_2_ transparent ceramic doped with Eu^2+^. Ceram. Int..

[22] Lan Y., Mei B., Li W., Xiong F., Song J. (2018). Preparation and scintillation properties of Eu^2+^:CaF_2_ scintillation ceramics. J. Lumin..

[23] McGregor D.S. (2018). Materials for gamma-ray spectrometers: Inorganic scintillators. Annu. Rev. Mater. Res..

[24] Zhou L., Chen D., Luo W., Wang Y., Yu Y., Liu F. (2007). Transparent glass ceramic containing Er^3+^:CaF_2_ nano-crystals prepared by sol–gel method. Mater. Lett..

[25] Krause W., Nolze G. (1996). PowderCell a program for the representation and manipulation of crystal structures and calculation of the resulting X-ray patterns. J. Appl. Cryst..

[26] Secu C.E., Predoi D., Secu M., Cernea M., Aldica G. (2009). Structural investigations of sol–gel derived silicate gels using Eu^3+^ ion-probe luminescence. Opt. Mater..

[27] Rüssel C. (1993). Thermal decomposition of metal trifluoracetates. J. Non-Cryst. Solids.

[28] Secu C.E., Bartha C., Polosan S., Secu M. (2014). Thermally activated conversion of a silicate gel to an oxyfluoride glass ceramic: Optical study using Eu^3+^ probe ion. J. Lumin..

[29] Luo W., Wang Y., Cheng Y., Bao F., Zhou L. (2006). Crystallization and structural evolution of SiO_2_-YF_3_ xerogel. Mater. Sci. Eng. B.

[30] Yu Y., Chen D., Wang Y., Luo W., Zheng Y., Cheng Y., Zhou L. (2006). Structural evolution and its influence on luminescence of SiO_2_–SrF_2_–ErF_3_ glass ceramics prepared by sol–gel method. Mater. Chem. Phys..

[31] Szpikowska-Sroka B., Zur L., Czoik R., Goryczka T., Swinarew A.S., Zadło M., Pisarski W.A. (2013). Long-lived emission from Eu^3+^-doped PbF_2_ nanocrystals distributed into sol–gel silica glass. J. Sol-Gel Sci. Technol..

[32] Del-Castillo J., Yanes A.C., Mendez-Ramos J., Tikhomirov V.K., Moshchalkov V.V., Rodrıguez V.D. (2010). Sol–gel preparation and white up-conversion luminescence in rare-earth doped PbF_2_ nanocrystals dissolved in silica glass. J. Sol-Gel Sci. Technol..

[33] Aguiar H., Serra J., Gonzalez P., Leon B. (2009). Structural study of sol–gel silicate glasses by IR and Raman spectroscopies *J*. Non-Cryst. Solids.

[34] Wang F., Fan X., Pi D., Wang M. (2005). Synthesis and luminescence behavior of Eu^3+^-doped CaF_2_ nanoparticles. Solid State Commun..

[35] Labéguerie J., Gredin P., Mortier M., Patriarche G., de Kozak A. (2006). Synthesis of Fluoride Nanoparticles in Non-Aqueous Nanoreactors. Luminescence Study of Eu^3+^: CaF_2_. Z. Anorg. Allg. Chem..

[36] Dorenbos P. (2003). Energy of the first 4f^7^→4f^6^5d transition of Eu^2+^ in inorganic compounds. J. Lumin..

[37] Kobaiashi T., Mroczkowski S.J., Owen F., Brixner L. (1980). Fluorescence lifetime in Eu^2+^-doped chlorides and fluorides *J*. Lumin..

[38] Dorenbos P., den Hartog H.W. (1985). Space charges and dipoles in rare-earth-doped SrF_2_. Phys. Rev. B.

[39] Silversmith A.J., Radlinski A.P. (1985). Zeeman spectroscopy of the G1 centre in CaF_2_:Eu^3+^. J. Phys. C Solid State Phys..

[40] Baker J.M., Bleaney B., Hayes W. (1958). Paramagnetic resonance of *S*-state ions in calcium fluoride. Proc. R. Soc. Lond.

[41] Antuzevics A., Kemere M., Krieke G., Ignatans R. (2017). Electron paramagnetic resonance and photoluminescence investigation of europium local structure in oxyfluoride glass ceramics containing SrF_2_ nanocrystals. Opt. Mater..

[42] Title R.S. (1963). The cubic field splitting of the Eu^2+^ EPR spectrum in the alkaline earth flourides. Phys. Lett..

[43] Secu C.E., Negrila C., Secu M. (2018). Investigation of sol-gel derived BaCl_2_:Eu^2+^ luminescent nanophosphor and the corresponding PVP@BaCl_2_:Eu^2+^ polymer nanocomposite. J. Phys. D Appl. Phys..

[44] Vercaemst R., Poelman D., Fiermans L., Van Meirhaeghe R.L., Laflère W.H., Cardon F. (1995). A detailed XPS study of the rare earth compounds EuS and EuF_3_. J. Electron Spectrosc. Relat. Phenom..

[45] Mercier F., Alliot C., Bion L., Thromat N., Toulhoat P. (2006). XPS study of Eu(III) coordination compounds: Core levels binding energies in solid mixed-oxo-compounds Eu_m_X_x_O_y_. J. Electron Spectrosc. Relat. Phenom.

[46] Bos A.J.J. (2017). Thermoluminescence as a Research Tool to Investigate Luminescence Mechanisms. Materials.

[47] Bos A.J.J., Dorenbos P., Bessière A., Viana B. (2008). Lanthanide energy levels in YPO_4_. Radiat. Meas..

[48] Krumpel A.H., van der Kolk E., Zeelenberg D., Bos A.J.J., Krämer K.W., Dorenbos P. (2008). Lanthanide 4f-level location in lanthanide doped and cerium-lanthanide codoped NaLaF_4_ by photo- and thermoluminescence. J. Appl. Phys..

[49] Secu C.E., Secu M., Ghica C., Mihut L. (2011). Rare-earth doped sol–gel derived oxyfluoride glass–ceramics: Structural and optical characterization. Opt. Mater..

[50] Alvarez Rivas J.L. (1980). Thermoluminescence and lattice defects in alkali halides. J. Phys. Colloq..

[51] Secu C.E., Rostas A.M. (2020). Investigations of BaCl_2_:Eu^2+^ nanophosphor using electron paramagnetic resonance, structural analysis and thermoluminescence. J. Alloys Compd..

[52] Secu M., Secu C.E. (2020). Processing and Optical Properties of Eu-Doped Chloroborate Glass-Ceramic. Crystals.

[53] Schweizer S., Hobbs L.W., Secu M., Spaeth J.-M., Edgar A., Williams G.V.M. (2003). Photostimulated luminescence in Eu-doped fluorochlorozirconate glass ceramics. Appl. Phys. Lett..

[54] Wintle A.G. (1975). Thermal Quenching of Thermoluminescence in Quartz Geophys. J. Int..

