# Supplementary material for: Europium (II)-Doped CaF2 Nanocrystals in Sol-Gel Derived Glass-Ceramic: Luminescence and EPR Spectroscopy Investigations
Source: Nanomaterials (Basel). 2022 Aug 31;12(17):3016. doi: 10.3390/nano12173016 (PMC9457762; doi:10.3390/nano12173016)
Supplement: Supplementary file 1 [file nanomaterials-12-03016-s001.zip › nanomaterials-1852485-supplementary.pdf]

Application Note BRUKER

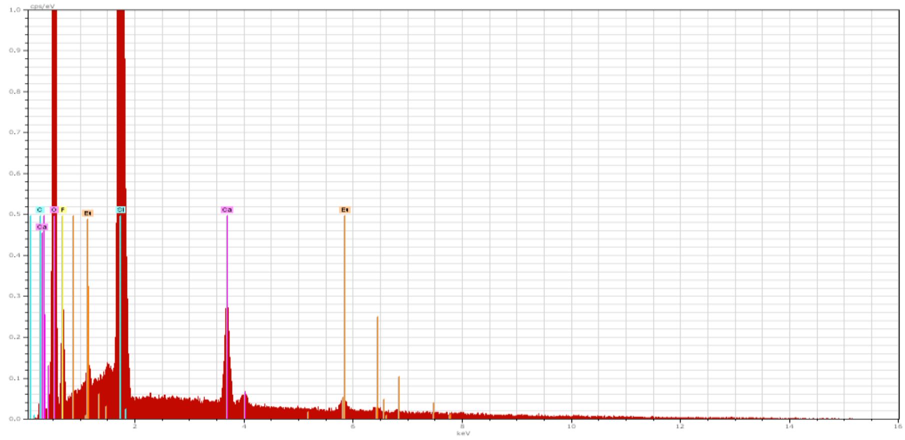

Spectrum: spectrum

El	AN	Series	unn. C [wt.-%]	norm. C [wt.-%]	Atom. C [at.-%]	Error [%]
C	6	K-series	1.34	1.13	1.93	0.3
O	8	K-series	47.89	40.49	51.84	5.5
F	9	K-series	18.21	15.39	16.60	2.9
Si	14	K-series	45.25	38.26	27.91	1.9
Ca	20	K-series	3.41	2.88	1.47	0.1
Eu	63	L-series	2.19	1.85	0.25	0.1
		Total:	118.29	100.00	100.00	

12/08/2022 Page 1 / 2

Figure S1. The EDX spectra analysis of the glass-ceramic sample.

The XPS spectra recorded on SiO_2-CaF_2-Eu glass-ceramic sample annealed in reducing atmosphere, shown in different energy regions

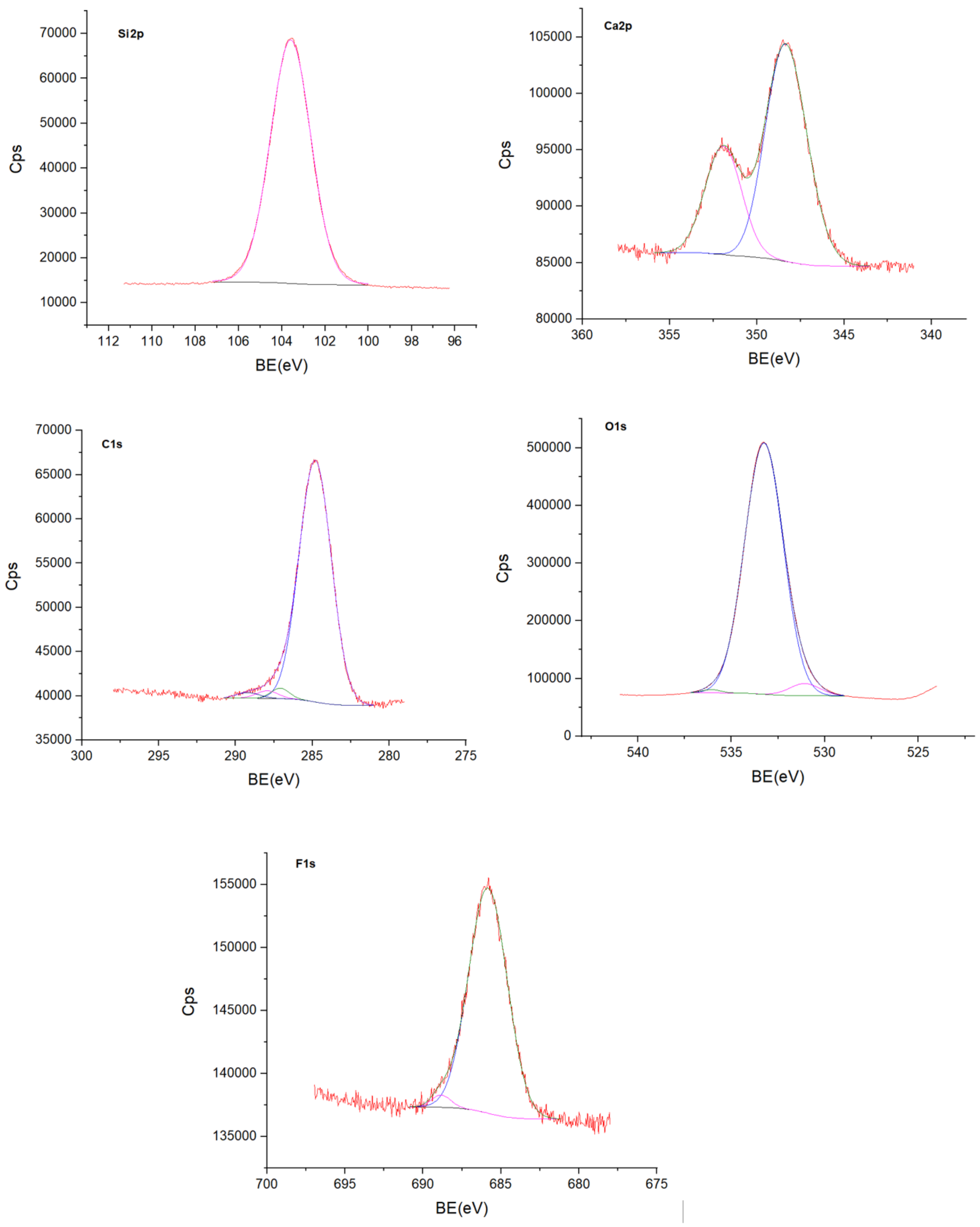

Figure S2. The XPS spectra were recorded on the glass-ceramic sample annealed in reducing atmosphere and shown in different energy regions.
